# Accurate Reading with Sequential Presentation of Single Letters

**DOI:** 10.3389/fnins.2012.00158

**Published:** 2012-10-30

**Authors:** Nicholas S. C. Price, Gemma L. Edwards

**Affiliations:** ^1^Department of Physiology, Monash UniversityMelbourne, VIC, Australia

**Keywords:** visual prosthesis, bionic vision, low vision, reading, RSVP, phosphene, word recognition

## Abstract

Rapid, accurate reading is possible when isolated, single *words* from a sentence are sequentially presented at a fixed spatial location. We investigated if reading of words and sentences is possible when single *letters* are rapidly presented at the fovea under user-controlled or automatically controlled rates. When tested with complete sentences, trained participants achieved reading rates of over 60 wpm and accuracies of over 90% with the single letter reading (SLR) method and naive participants achieved average reading rates over 30 wpm with greater than 90% accuracy. Accuracy declined as individual letters were presented for shorter periods of time, even when the overall reading rate was maintained by increasing the duration of spaces between words. Words in the lexicon that occur more frequently were identified with higher accuracy and more quickly, demonstrating that trained participants have lexical access. In combination, our data strongly suggest that comprehension is possible and that SLR is a practicable form of reading under conditions in which normal scanning of text is not possible, or for scenarios with limited spatial and temporal resolution such as patients with low vision or prostheses.

## Introduction

Worldwide, over a dozen teams are developing visual prostheses, which aim to return some visual sensitivity to blind people by electrically stimulating the retina (Argus II Retinal Prosthesis System, Second Sight Medical Products Inc “Argus II”; Dowling, 2009; Zrenner et al., 2011), lateral geniculate nucleus in the thalamus (Pezaris and Reid, 2007; Pezaris and Eskandar, 2009), or primary visual cortex (Brindley and Lewin, 1968; Dobelle and Mladejovsky, 1974; Dobelle et al., 1974; Schmidt et al., 1996; Bradley et al., 2005; Tehovnik and Slocum, 2007). Localized stimulation in any of these regions along the early visual pathway can produce the percept of a small patch of light, referred to as a phosphene. Therefore, simultaneous stimulation at multiple locations via spatially separated electrodes can be used to construct an image (Dobelle et al., 1976; Humayun et al., 1999).

Three primary functional and practical benchmarks for a visual prosthesis are to enable unassisted navigation, to simplify object manipulation, and to facilitate object and shape recognition. Shape recognition includes identifying letters and words, which are the critical first steps to enable reading. Although reading is likely to be one of the most difficult goals to achieve with a visual prosthesis, it is highly desired and valued by people with low vision (Massof, 1998; Hazel et al., 2000). While patients currently implanted with visual prostheses can identify letters and short words (da Cruz et al., 2010; Stanga et al., 2010), their performance is limited and accurate recognition requires significant time and mental effort. Therefore, simulations of prosthetic reading in normally sighted participants may allow extensive exploration and refinement of device requirements and possibilities. All previous approaches to simulating prosthetic reading have required converting a high resolution video signal into a continually changing, but low spatial resolution pattern of electrical stimulation, where each pixel in the simulated image will correspond to an electrically generated phosphene (Cha et al., 1992; Sommerhalder et al., 2003, 2004; Dagnelie et al., 2006; Fu et al., 2006). Thus, these prosthetic reading methods require scanning small patches of text by moving the video camera (i.e., with the head or eyes). While this is similar to the eye movements that occur during normal reading, continual movements present three primary hurdles for reading with a visual prosthesis, associated with contrast polarity, spatial resolution, and temporal resolution.

First consider contrast polarity; if text on screen or on paper (which is normally shown as black letters on a white background) were to be represented using a prosthesis this would require the majority of electrodes to be activated to represent the white background. This has the undesirable effects of increasing current leakage and power consumption in the device. Second, spatial resolution, or the number of pixels in the artificial image, is limited by the number of electrodes in the prosthetic device, with most systems expected to have just 60–1500 electrodes (Argus II; Zrenner et al., 2011). Most studies aim to use at least 600 electrodes to represent approximately four letters, since this allows relatively normal reading speeds (Legge et al., 1985; Cha et al., 1992; Sommerhalder et al., 2003). This is problematic, as a representation of four letters can only be achieved if the camera can be moved or zoomed so that its field of view closely matches the size of the text. Finally, temporal resolution is likely limited by both engineering and physiological considerations to as little as 2–20 Hz, which is lower than normal video refresh rates (Dobelle et al., 1976; Dobelle, 2000; Perez Fornos et al., 2010; Zrenner et al., 2011). However, previous simulations have assumed that the prosthetic image is updated at video rates of higher than 30 Hz, where motion blur is not a problem (Cha et al., 1992; Sommerhalder et al., 2003; Dagnelie et al., 2006; Fornos et al., 2011).

**Figure 1:**
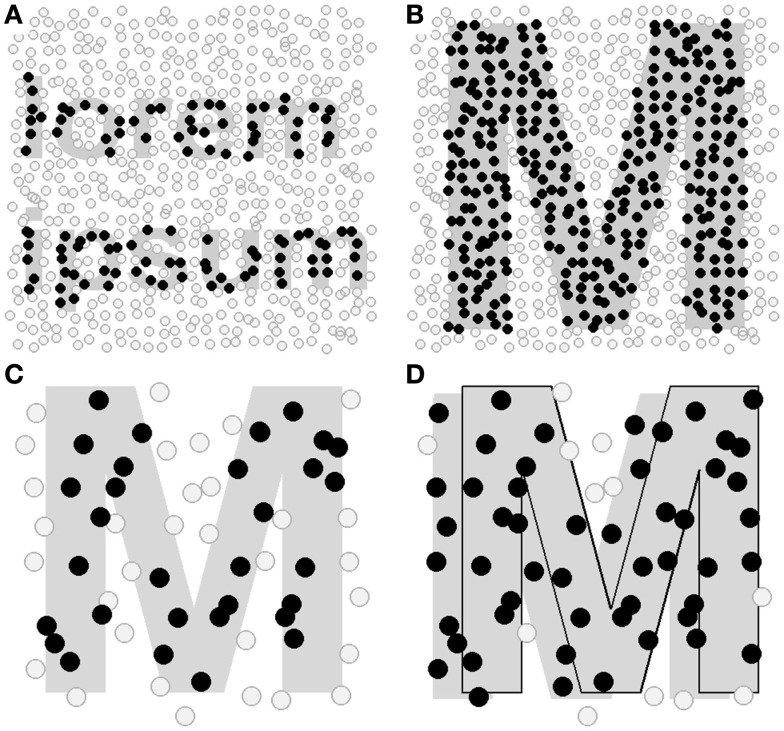
**Representation of *lorem ipsum* (A) and M (B) by a random grid of 25 × 25 “phosphenes.”** Circles show the phosphene locations and are filled to show the phosphenes that would be activated to represent the stimulus shown by the gray mask. Only the black circles, not the gray mask or gray circles, would be evident in the prosthetic image. In a single frame, the two words *lorem ipsum* cannot be reliably represented, but the single letter M is easily represented. Note that if the camera (i.e., the patient’s eye or head) moves with respect to the text, different sets of electrodes will be activated, making some phosphenes turn on and off. With an 8 × 8 grid of phosphenes, stable single letters can be reliably represented **(C)**, however, if the letters are not stabilized with respect to head or eye movements, even single letters may become unrecognizable. In **(D)**, the M has been replicated and overlaid to the right by 10% of its width, mimicking motion blur due to a small camera movement. As the majority of electrodes would now be activated, it is difficult to identify the letter from this pattern. Therefore presenting a single spatially stabilized letter is the ideal compromise when spatial resolution is limited.

Based on the high reading rates achievable with Rapid Serial Visual Presentation (RSVP) of isolated words (Gilbert, 1959; Forster, 1970), we wondered if similar presentation methods would facilitate fast, accurate reading with a visual prosthesis. While simultaneously rendering an entire word with a few hundred randomly placed phosphenes is extremely difficult, rendering a single letter is simple (Figure 1). Here, we designed a simulation for normally sighted people that allows us to evaluate a novel Single Letter Reading (SLR) method for use with bionic eyes. In contrast to previous RSVP methods which present a whole word at a time, we use rapid, sequential presentation of single *letters* in a fixed foveal location. Reading accuracy and overall reading rate were assessed as we systematically varied font size, the presentation durations of individual letters, gaps between letters, and spaces between words and the degree of user control of letter, and word presentation. When tested with isolated words and complete sentences, normally sighted, trained participants demonstrated lexical access and achieved reading rates of over 60 wpm and accuracies of over 90%. Naive participants with no previous exposure to SLR achieved average reading rates over 30 wpm and over 90% accuracy within a single testing session. While the lexical access, reading rates and accuracies we have observed should be sufficient to allow accurate comprehension (Pelli et al., 1985; Whittaker and Lovie-Kitchin, 1993; Coltheart et al., 2001), this was not directly assessed. Therefore, future work will examine how different presentation rates and methods affect the comprehension of longer passages of text. We anticipate that SLR will facilitate accurate and efficient reading with any prosthetic visual device, as the method is not greatly affected by limitations in spatial or temporal resolution.

## Materials and Methods

### Stimuli

Stimuli were generated using Matlab (The Mathworks, Natick, MA, USA) and the Psychophysics Toolbox extensions (Brainard, 1997; Pelli, 1997) and were viewed binocularly on a Sony Multiscan G500 monitor (1280 × 960 pixels; viewable area 400 × 300 mm; refresh rate 100 Hz). Head position was not stabilized, as reading aloud with a chin rest is difficult, but a viewing distance of 600–700 mm was maintained. Stimuli comprised whole words, presented in white Arial font with a vertical span of 25–200 pixels for a capital letter (0.65–5.9°). Importantly, apart from control conditions, only a single letter was visible at a time, with all letters presented sequentially in the same location at the middle of a black screen.

In different tasks, participants read aloud single words, non-words, whole sentences or 100–101 word short stories. In the majority of tasks, only a single stationary letter was visible at any time, a general method referred to here as SLR. For single word stimuli, two presentation methods were used to govern the temporal display of individual letters: Elicited Sequential Presentation of Letters (ESP-Letters) and Automated Sequential Presentation of Letters (ASP-Letters). In ESP-Letters, participants pressed a button to display the first letter, which remained on the screen until the next button press displayed the subsequent letter in its place and so on. In ASP-Letters, individual letters were visible for 20–390 ms (*t*_visible_), separated by a inter-letter gap of a 10–20 ms blank screen (*t*_gap_). All letters within a word were automatically presented for the same duration (*t*_letter_ = *t*_visible_ + *t*_gap_). ASP-Letters is the letter-based equivalent of the commonly used RSVP method of presenting whole words. ESP-letters is the letter-based equivalent of the ESP method of presenting whole words at a time (Arditi, 1999). For sentence stimuli, the individual letters within a word appeared with automated timing (i.e., ASP-Letters), however, the start of each word could either be initiated by a button press (ESP-Words) or could occur with preset timing (ASP-Words) with 20–1500 ms blank screen between words (*t*_space_). Specific details of each task are given below.

### Text sources

Single words were extracted from the English Lexicon Project (ELP) Database at http://elexicon.wustl.edu/ (Balota et al., 2007). Only common, high frequency words were used to ensure that participants were familiar with the majority of words. High frequency words have the advantage that they are better recognized, produced, and recalled (Brysbaert and New, 2009). Word frequencies were determined from the Hyperspace Analog to Language (HAL) corpus of over 400 million words from all textual Usenet newsgroups (online discussion forums) during February 1995 (Lund and Burgess, 1996; Balota et al., 2007). The HAL corpus is much larger than the commonly used Kučera and Francis corpus (Kučera and Francis, 1967), giving more reliable estimates of word frequency. Our measure of word frequency is the log of the HAL frequency, which is simply the log of the number of occurrences of a given word in the HAL corpus. Some example words of different lengths and their associated log HAL frequencies are: 15 – *be, this*; 10 – *rob, honest, dangerous*; 5 – *mew, accede, captaincy*; 2 – *gyp, tousle, ambrosial*.

Non-words were downloaded from the ARC Non-word Database http://www.maccs.mq.edu.au/∼nwdb (Rastle et al., 2002). Only monomorphemic pseudohomophones with orthographically existing onsets and bodies, and legal bigrams were used. Monomorphemic words contain only a single grammatically or semantically meaningful unit, such as “like” and “trouble,” but not “dislike” and “troublesome” which have two morphemes. No bound morphemes (e.g., un-, dis-, -ed, -ing) were included. A pseudohomophone is a non-word that is phonetically identical to a real word. Thus, all the non-words used here contain letter pairings that look and sound like parts of real words (e.g., *grone* which is pronounced like *groan*).

Isolated sentences were sourced from 18 fictional eBooks freely available on the Internet. Unwanted characters and words such as page numbers and chapter headings were automatically removed and subsequently checked manually. To ensure that all words could be linguistically analyzed, sentences containing numbers, symbols, abbreviations, or words that were not in the ELP database were removed. Only sentences with 4–12 words were used, with no word exceeding 12 letters in length. The final stimulus pool contained 18,852 complete sentences. With all sources, participants were warned that words would appear with American spelling.

Short stories (100–101 words) were sourced from two websites; http://joecliffordfaust.com/category/100-word-short-stories/ (where all the short stories were written by the one published science-fiction author), and http://www.101words.org/ (where anyone can submit a story). All stories from these sites were manually screened and stories containing quotation marks or content deemed inappropriate to read aloud in a lab setting were excluded. Subsequently, stories were edited, a process that included: converting numbers from numerals to words; converting spellings to Australian English; ensuring that all words passed a spell check in Microsoft Office Word 2007; expanding uncommon abbreviations (e.g., DA to “District Attorney”) and changing uncommon proper nouns to common names. Grammar, HAL frequency, the number of words in a sentence, and the number of sentences in a story were not considered. Punctuation was retained.

### Single word reading task

The single word task required participants to view and read aloud one word at a time. Only words from the ELP database with a log HAL frequency above 9.2 were used. Blocks of 72 words were tested, with each block containing eight different words of 3–11 letters length. Words were presented in random order, with no intentionally meaningful groups or sequences. Within a block, only one presentation type was used; either ESP-Letters or ASP-Letters. With ESP-Letters, letters remained on the screen until the participant pressed a key to view the next letter. With ASP-Letters, two stimulus sets were employed to systematically determine the effects of letter timing and font size on reading accuracy. The first stimulus set primarily explored timing; *t*_visible_ changed randomly every eight words (50–400 ms), but *t*_gap_ was always 10 ms. A fixed font size of 40 pixels was used. Word length and word frequency were not balanced within a sub-block of eight trials with the same *t*_visible_. In the second ASP-Letters stimulus set, all words in a block had the same font size (25–200 pixels), with a limited range of timings (*t*_visible_ = 20–160 ms across blocks; *t*_gap_ = 20 ms). In pilot testing, participants reported that the longer *t*_gap_ (20 versus 10 ms) was easier to read, but this effect of timing was not quantified or extensively explored.

Participants were allowed to practise both ESP-Letters and ASP-Letters reading until they were comfortable with each technique. Subsequently, they completed 360 ESP-Letters trials and 672-4120 ASP-Letters trials. On each trial, participants silently viewed a letter string followed by “?” cueing them to respond by reading the perceived word aloud. They were encouraged to aim for accuracy not speed, but generally responded within 1 s. If unsure about a word, participants could guess or simply say “I don’t know.” Verbal responses were recorded directly to MP3 files and subsequently manually transcribed for scoring. Words were only scored as correct if the presented and spoken words matched perfectly (e.g., the response “cat” was incorrect if “cats” was presented). Reaction times were not analyzed.

Four participants completed the ESP-Letters task and four participants completed the ASP-Letters task. Three participants completed both tasks.

### Lexical decision task

Along with accurate identification of words, “lexical access” is a necessary step toward reading comprehension. Lexical access refers to the process of determining whether a sequence of letters belongs to the mental lexicon and locating the word within this organized vocabulary (Field, 2003). To determine if participants have lexical access we used a commonly employed word versus non-word lexical decision task (LDT) in which three participants categorized strings of letters as real words or non-words. The classical result in such a task is that words are correctly classified more quickly than non-words, and words that occur more frequently are more rapidly identified as true words (Forster and Chambers, 1973). Collectively, these result suggest that lexical access has occurred. Real words were extracted from the ELP database and were three to seven letters in length, had a log HAL frequency greater than six and were monomorphemic. Only common nouns, verbs, adjectives, and adverbs were used. Non-words were extracted from the ARC non-word database (see Text Sources above) and were three to seven letters in length. All letter strings were presented using the ASP-Letters method with a fixed font size of 45 pixels. In different blocks, [*t*_visible_ *t*_gap_] pairings of [180 20], [20 180], [80 20], [20 80] ms were employed.

Each participant completed 3–11 blocks of trials at each letter timing, with each block containing 30 real words and 30 non-words in random order. On each trial, participants silently viewed a letter string followed by “?” cueing them to press a button to indicate whether the string was a real word or non-word. They were asked to aim for accuracy rather than speed, but reaction times were recorded relative to the disappearance of the last letter and were limited to 1.75 s. Reaction times were only compared between words and non-words with the same length, ensuring equal viewing times. As participants can begin lexical processing of the word during word presentation, we only examine relative, not absolute reaction times. To reduce priming effects, each participant never saw the same word twice in training or testing.

### Sentence reading task

The sentence task tested three participants’ ability to read sentences of 4–12 words using the SLR method with a font size of 50 pixels. Each participant viewed and read aloud at least 480 sentences (greater than 3000 words), with their responses recorded for offline scoring. Across a block of 10 sentences, the same timing conditions were used for all words, and ASP-Letters (*t*_visible_ = 20, 40, 80, or 120 ms; *t*_gap_ = 20 ms). Two word presentation methods were tested: ESP-Words, in which a blank screen was shown between words, with a keypress required to initiate presentation the next word; and ASP-Words, in which an entire sentence was presented with automated timing. For ESP-Words, only one timing parameter was varied – *t*_visible_, which controls the duration for which each letter is visible. For ASP conditions, two parameters were varied: *t*_visible_ and *t*_space_, which controls the duration of blank screen between words.

In testing, each participant first read 30–40 sentences with ESP-Words, with each of the four *t*_visible_ timing conditions. For each participant and each condition, the average time between the end of one word and the keypress to elicit the next word was determined (*t*_space, ESP_). This was used to set individualized slow, medium/mid, and fast presentation rates for use in the ASP-Words conditions. In the mid-ASP condition, *t*_space_ was set to give the same reading rate as each participant’s average ESP-Words reading rate. The fast- and slow-ASP conditions had *t*_space_ values scaled by 2/3 and 3/2 relative to the average ESP rate (*t*_space, ESP_). Each participant then read 30–40 sentences with each of the 12 ASP-Words conditions (three values of *t*_space_ * four values of *t*_visible_).

Participants were asked to aim for accuracy and could correct themselves. However, as we are interested in baseline reading accuracy, for simplicity, we assess each participant’s first impression of a word and ignore any corrections. Participants could read each sentence aloud as the words were presented, read a few words in a cluster, silently view all words and then say the entire sentence aloud, or a combination of these; essentially, participants were asked to read aloud in any way that seemed natural and easy. When presentation rates were slow, it is straightforward to read aloud each word as it is presented, however, at fast presentation rates, participants generally switched to a strategy of reading clusters of words at a time or reading an entire sentence from memory.

### Paragraph reading task

This task involved participants reading aloud short stories of 100–101 words as they were presented on the screen sentence-by-sentence, word-by-word, or letter-by-letter. Stories were randomly allocated to each of these presentation methods, then all participants read each story in the same order.

In all cases, participants were asked to aim for accuracy in their responses. Before testing of a particular condition commenced, participants were shown the stimulus, had the task briefly demonstrated to them by the experimenter, and were given a chance to practise reading words. In the first control condition, all words within a sentence were presented simultaneously on the screen and the participant pressed a key to view the next sentence. In the second control, single words were presented and the participant pressed a key to view the next word. In the final two SLR conditions, the ESP-Words method was used with *t*_visible_ = 80 or 120 ms and *t*_gap_ = 20 ms (i.e., *t*_letter_ = 100 or 140 ms). Each participant viewed three to four stories with each presentation condition, proceeding from the Sentence control condition, to the Word control condition, to the SLR conditions; first ESP-Words with *t*_letter_ 140 ms and subsequently with *t*_letter_ 100 ms. Stories were viewed in the same order and no participant was ever tested on the same story twice.

In the SLR conditions, participants could repeat a word by pressing the left key, or proceed to the next word by pressing the right key. To prevent participants from repeatedly viewing difficult words, each word could only be repeated twice (a total of three viewings). Due to a programming error, participants could not always repeat the very first and last words of the story. As participants could review and re-attempt words, their last response to each word was the one that was scored as accurate or inaccurate (note that in the Sentence Reading Task, only the first utterance of a word was scored). Participants were not penalized for context-dependent mispronunciations, e.g., the word “live” has two pronunciations, rhyming with “give” and “hive,” but both pronunciations were scored as correct. Reading accuracy was defined as the percentage of correctly identified words. Reading rate was defined as the total number of words in a story divided by the time taken to read aloud all words.

### Participants

Participants (ages 20–33; eight female, three male) were recruited from within the Department of Physiology at Monash University and gave informed consent. All had English as a first language and normal or corrected-to-normal vision. All scoring was performed by participants S3, S4, and S5 (who also scored themselves).

For the single word reading task, LDT and sentence reading task, participants were the authors and three undergraduate research assistants who were compensated for their time. Two participants completed all testing requirements (approximately 25 h, not including breaks, training, and pilot testing) over 9 months. Three participants completed a subset of the tests. Participants were aware of the motivations, details of the presentation parameters, and their performance throughout the task. For the paragraph reading task, participants included two trained observers from the first cohort who were compensated for their time (S4 and S5) and six naive, uncompensated volunteers with little or no psychophysical testing experience. Participants were not informed of their performance throughout the task.

## Results

### Reading single words

**Figure 2:**
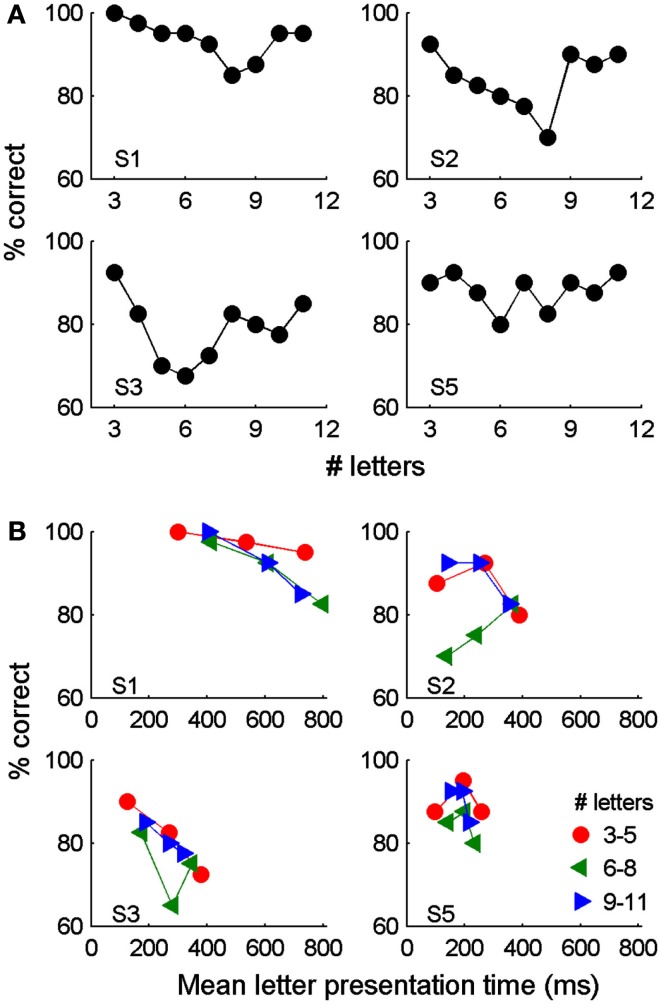
**Reading of isolated words using the ESP-Letters method in which participants determined the rate of letter presentation within each word**. Reading accuracy as a function of word length **(A)** and mean letter presentation time **(B)** are shown separately for four participants, who each viewed a total of 360 words. In **(B)**, each data point represents the mean performance on trials from each tercile of letter presentation times. As longer words take longer to present, words were also grouped by length.

The ability of participants to accurately read isolated words was initially tested by giving participants complete control over the rate at which letters appeared (i.e., ESP-Letters). While one participant correctly identified all three letter words, no participants correctly identified words of all possible lengths and the average performance across participants was only 86%. Performance within individual participants did not change significantly across word lengths of 3–11 letters [Figure 2A; Pearson chi-squared test χ^2^(8, 360)_S1_ = 11.8; χ^2^(8, 360)_S2_ = 12.2; χ^2^(8, 360)_S3_ = 12.0; χ^2^(8, 360)_S5_ = 5.6. *p* > 0.05 for all participants]. With ESP, presentation rates are somewhat limited by how rapidly the participant can press a button to see the next letter. In most trials, participants converged on a technique of rhythmically pressing a button to view each letter, with average presentation rates of less than three letters/second. We hypothesized that performance might be better on trials in which presentation rates were slower. To assess this, for groups of words with similar lengths we created terciles of letter presentation time and found the average performance for each grouping. Average letter presentation duration was not systematically related to performance for any participant or word length (Figure 2B), but there is a surprising trend toward higher accuracies with faster reading speeds, especially for longer words. This goes against the expected speed-accuracy trade-off and may be because longer words may have few orthographic neighbors and thus can be uniquely identified by their first few letters. This would allow an increased rate of letter presentation after a word is identified (e.g., the sequence “e-l-e-p” is sufficient to predict “elephant”). Alternatively, the poor performance at long presentation durations may arise because it is attentionally demanding to attempt to read at such abnormally slow rates.

**Figure 3:**
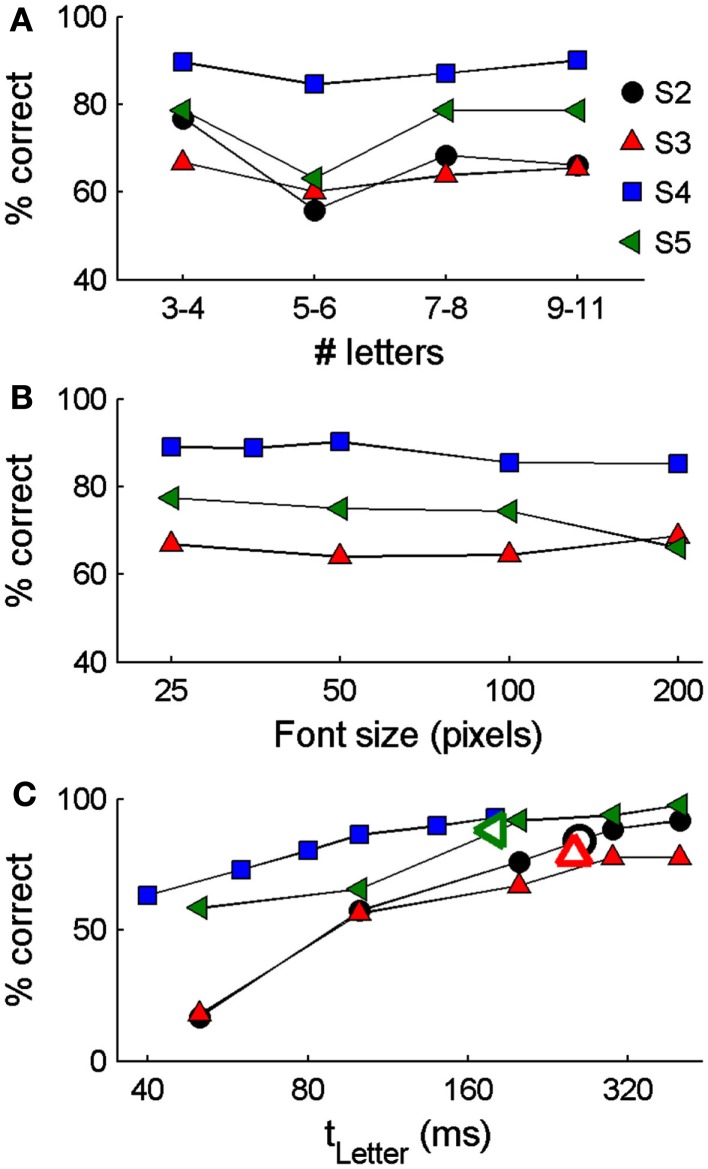
**Single word reading performance with the ASP-Letters method in which the presentation rate of letters in a word is automated**. Results are shown separately for words of different lengths **(A)**, font sizes **(B)**, and letter durations **(C)**. Subjects were tested with different conditions: in **(A)**, only presentation times (*t*_letter_) 80–200 ms and font sizes of 50–100 pixels were included; in **(B)**, all tested word lengths (3–11 words) and letter timings of 80–200 ms were included; in **(C)** all word lengths and font sizes of 50–100 were included. For subjects S3 and S4, the inter-letter blank duration *t*_gap_ was 20 ms. For subjects S2 and S5, *t*_gap_ was 10 ms. Open markers in **(C)** indicate the median letter presentation times and overall percent correct using ESP-Letters for S2, S3, and S5.

Based on the observation that participants often adopted a regular rhythmic viewing strategy and that performance was relatively independent of presentation rate when using ESP-Letters, we examined how performance depended on letter timing using ASP-Letters. Blocks of 8 to 64 trials had the same timing, so that participants knew the speed with which letters would appear. Average performance across all four participants ranged from 60 to 90% (Figure 3A). As with ESP-Letters, performance in the ASP-Letters condition performance did not depend on word length for three of four participants [Pearson chi-squared test χ^2^(3, 192)_S2_ = 4.3, *p* = 0.23; χ^2^(3, 2456)_S3_ = 6.7, *p* = 0.08; χ^2^(3, 336)_S4_ = 8.0, *p* = 0.24; χ^2^(3, 960)_S5_ = 4.2, *p* = 0.05]. In the three participants tested with different font sizes, performance was not significantly affected by font size, across three octaves of size variations [Figure 3B, Pearson chi-squared test χ^2^(3, 3288)_S3_ = 4.2, *p* = 0.24; χ^2^(3, 672)_S4_ = 6.3, *p* = 0.06; χ^2^(4, 2400)_S5_ = 9.1, *p* = 0.10].

Importantly, performance depended significantly on letter presentation duration [Figure 3C; for all participants, *p* < 0.001, Pearson chi-squared test χ^2^(4, 432)_S2_ = 114; χ^2^(4, 504)_S3_ = 73; χ^2^(4, 216)_S4_ = 38.0; χ^2^(5, 4120)_S5_ = 306). Further, for participants two, three, and four, reading accuracy improved significantly as letter duration was increased (*p* < 0.05, Cochran–Armitage chi-squared test for departure from linear trend). Note that this is the opposite trend to that observed with ESP (Figure 2B). Even at the slowest presentation rates, 100% performance was not reached (Figure 3C). At the median ESP-Letters rate (190–260 ms/letter), accuracy under ESP-Letters and ASP-Letters conditions was similar (Figures 2A and 3C), but the maximum possible reading rate with ESP appears limited by how rapidly a participant can press a button.

### Lexical access is possible with SLR

**Figure 4:**
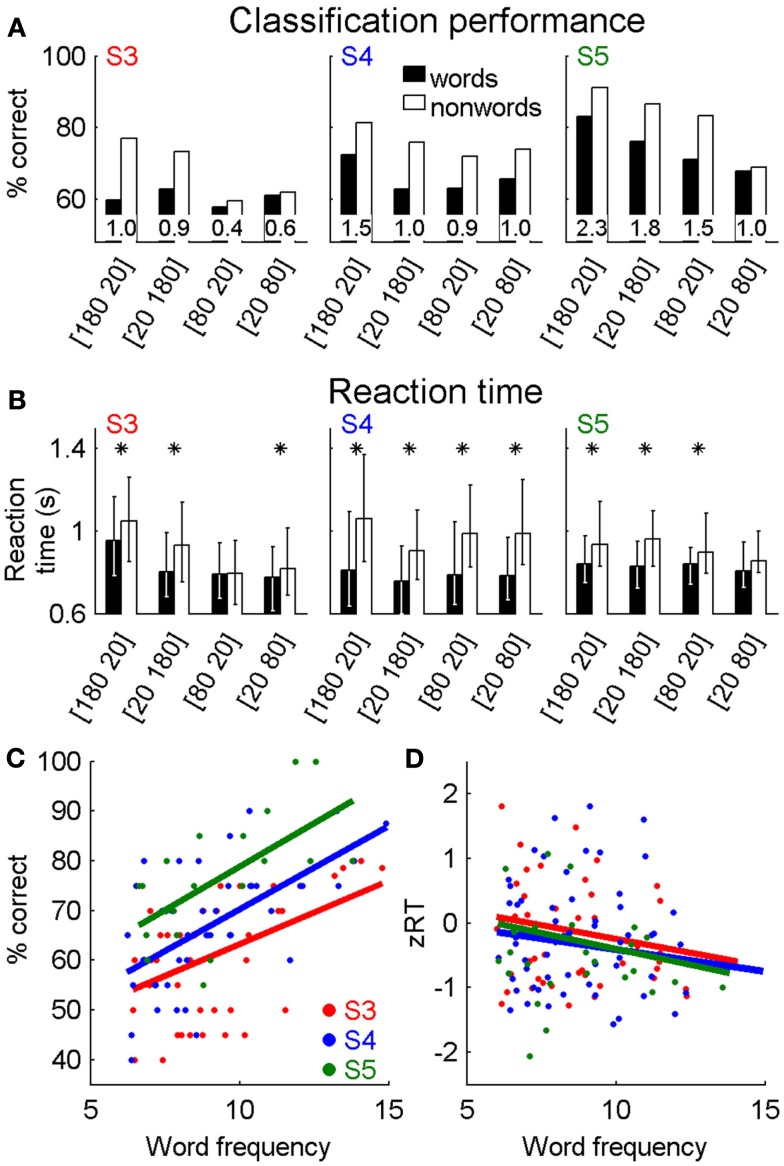
**Lexical decision task**. Words and non-words were correctly identified at higher than chance levels **(A)** with *d*′ > 0 for each timing condition (*d*′ values are inset within each pair of bars). The number pairs indicate the timing of letter presentation [*t*_visible_ *t*_gap_]. Median reaction times were typically shorter for correctly identified words than non-words **(B)**, with asterisks indicating median reaction times that are significantly different (rank-sum test, *p* < 0.05). For true words, word frequency (log HAL frequency) was positively correlated with accuracy **(C)** and normalized reaction times [*z*RT, **(D)**]. Data is based on judgments of 1596 (S3), 1748 (S4), and 720 (S5) words and non-words.

In the LDT, three participants classified monomorphemic words and pseudohomophonic non-words as words or non-words. Across a range of letter presentation timings, performance was above chance, and *d*′ values were positive for all participants (Figure 4A). The proportion of correctly classified non-words was higher than that of words. Of critical importance in a lexical task was the finding that the average reaction times for correct classification trials were significantly longer for non-words than words for the majority of timing conditions (rank-sum test, *p* < 0.05; asterisks in Figure 4B).

For real words, we compared classification performance and reaction time with each word’s HAL frequency (Figures 4C,D). As performance for individual words is either correct or incorrect, words were sorted by HAL frequency and grouped into blocks of 20 words with adjacent frequencies to find classification performance across a block. Reaction times were normalized by *z*-scoring the data separately for each participant and stimulus condition (e.g., data from four letter words with 200 ms/letter were normalized separately from five letter words with 200 ms/letter and also from four letter words with 100 ms/letter). In all participants, classification performance was positively correlated with log HAL frequency (*r*_S3_ = 0.46, *r*_S4_ = 0.61, *r*_S5_ = 0.62; *p* < 0.01 for all participants), and reaction time was negatively correlated with log HAL frequency (*r*_S3_ = −0.17, *r*_S4_ = −0.13, *r*_S5_ = −0.17; *p* < 0.01 for all participants), indicating that words occurring more frequently in the lexicon were more accurately classified, and were also identified more quickly.

We compared the reaction times for correctly identified words in our task with those available for a LDT in the ELP database (Balota et al., 2007). For the timing condition (*t*_visible_ = 180 ms *t*_gap_ = 20 ms), which produced the largest differences in performance and reaction times across participants, reaction times of two participants were significantly negatively correlated with those in the larger ELP database (*r*_S3_ = −0.22, *p* = 0.02; *r*_S4_ = −0.16, *p* = 0.03; *r*_S5_ = −0.21, *p* = 0.07).

### Reading sentences with SLR

**Figure 5:**
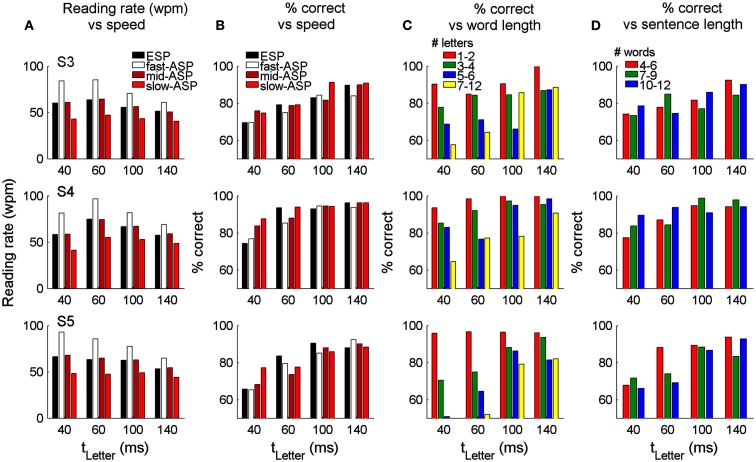
**Reading performance with whole sentences presented using ESP-Words (participant controlled rate) and ASP-Words (computer controlled rate)**. Reading rate **(A)** and percent of correctly identified words **(B)** for four presentation conditions and four letter timings (40–140 ms). In the ESP-Words condition, the timing of the appearance of each word was under user control. The average inter-word timing defined the automatic inter-word timing used in the mid-ASP-Words mid condition. Inter-word timings in the fast and slow-ASP-Words conditions were 2/3 and 3/2 of the mid-ASP-Words rate, respectively. **(C)** Word identification performance as a function of word lengths **(C)** and sentence lengths **(D)** for the mid-ASP-Words condition.

To test reading of whole sentences, we initially measured reading rates and accuracy with the ESP-Words condition, in which participants controlled the onset of each word, but the timing of individual letters was under automatic control (i.e., ASP-Letters with *t*_letter_ = 40–140 ms, with *t*_gap_ fixed at 20 ms). Regardless of letter timing, reading rates were quite constant (black bars in Figures 5A,B), varying from 51–75 wpm across participants. Reading rate did not systematically vary with letter timing because participants adopted regular button pressing rates to view successive words, giving them the same total time to linguistically process each word. With *t*_letter_ = 100 ms, reading performance for all participants was ∼90% correct at ∼60 wpm. Critically, as with the single word task, reading performance was significantly affected by letter presentation durations [*p* < 0.05, Pearson chi-squared test, χ^2^(3, 1379)_S3_ = 50.8; χ^2^(3, 1161)_S4_ = 87.6; χ^2^(3, 1348)_S5_ = 87.1]. Further, longer letter presentation durations were associated with higher accuracy in S4 and S5 (*p* < 0.05, Cochran–Armitage chi-squared test for departure from linear trend). This suggests that under SLR conditions, reading rate can be manipulated by changing word presentation rates, while accuracy can be independently manipulated by varying letter presentation timing.

Although only three participants completed both the single word reading task and sentence reading tasks, we compared their performance in the two tasks under comparable ASP-Letters rates. For single words of all lengths and *t*_letter_ = 100 ms, performance was 56.2 (S3), 86.2 (S4), and 65.2% (S5) with ASP-Letters. With the same letter timing, performance in the ESP-Words Sentence Reading task was 82.9 (S3), 92.9 (S4), and 90.5% (S5). Although all participants showed clear improvement between tasks, we are unable to distinguish the origin of this improvement. All participants completed the single word tasks before starting the sentence task, thus the improvements could arise due to the benefit of training or the contextual benefit provided by sentences.

As the button pressing in the ESP-Words condition may interfere with lexical processing, or limit reading speed, the time between words (*t*_space_) was predetermined in ASP-Words to give three conditions for each letter timing (slow-, mid-, and fast-ASP-Words). In the mid-ASP condition, *t*_space_ was set to give the same reading rate as each participant’s average ESP-Words reading rate. The fast- and slow-ASP conditions had *t*_space_ values scaled by 2/3 and 3/2 relative to the ESP-Words rate. The resulting reading rates are shown in Figure 5A. Note that under ASP-Words, the reading rate is entirely determined by *t*_letter_ and *t*_space_, thus participants have no control of the speed of word presentation. Reading accuracy was significantly affected by *t*_space_ (i.e., the reading rate) only for the shortest letter presentation times (*t*_letter_ 40–60 ms) and was not significantly different between ESP-Words and the mid-ASP-Words condition, nor between the mid- and fast-ASP conditions (*p* > 0.05, Pearson chi-squared test, Figure 5B). While this demonstrates that automated presentation of individual words at rates comparable to those used in ESP-Words does not affect reading accuracy, all participants found the ASP-Words conditions more attentionally demanding than ESP-Words, and were more likely to make errors in identifying sequential blocks of words within a sentence.

**Figure 6:**
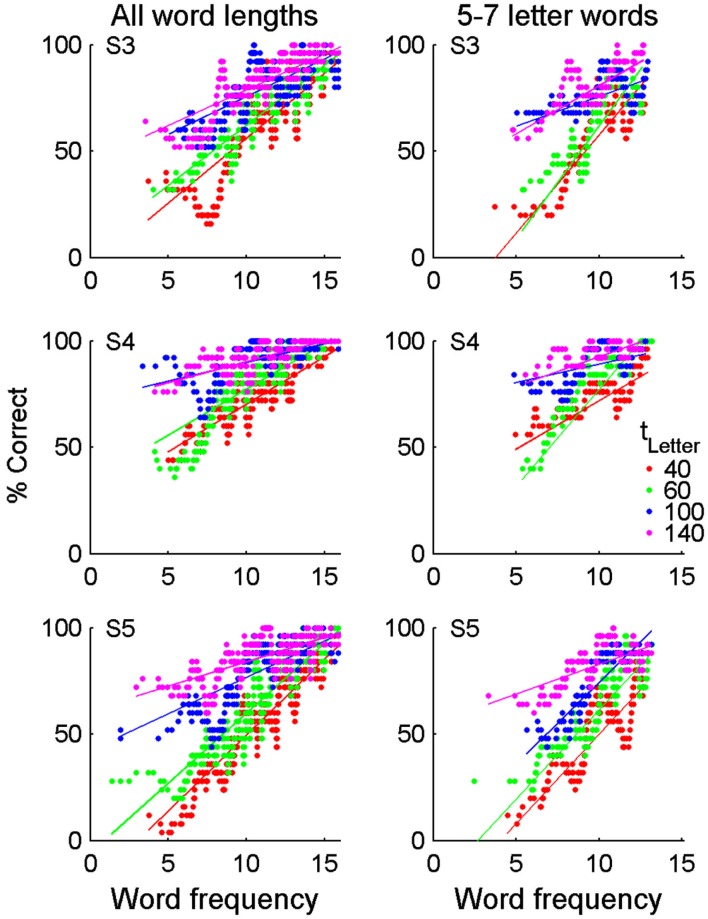
**Reading accuracy correlates with word frequency**. Words were sorted by their log HAL frequency and then reading accuracy was calculated in a sliding window across groups of 25 words with adjacent frequencies. Each data point thus represents reading accuracy of 25 words with similar frequency. Data is shown for all word lengths (1–12 letters, left column) and restricted to words of 5–7 letters (right column). Restricting the word length removes the confound associated with short and long words having the highest and lowest frequencies, respectively. Correlations between % correct and word frequency were significant (*p* < 0.01) when all word lengths were grouped: S3 – *r*_40_ = 0.37, *r*_60_ = 0.37, *r*_100_ = 0.27, *r*_140_ = 0.28; S4 – *r*_40_ = 0.32, *r*_60_ = 0.38, *r*_100_ = 0.21, *r*_140_ = 0.25; S5 – *r*_40_ = 0.43, *r*_60_ = 0.46, *r*_100_ = 0.26, *r*_140_ = 0.22. For words of 5–7 letters, correlations were only significant (*p* < 0.05) for the 40, 60, and 140 ms presentation rates: S3 – *r*_40_ = 0.32, *r*_60_ = 0.38, *r*_100_ = 0.19, *r*_140_ = 0.20; S4 – *r*_40_ = 0.19, *r*_60_ = 0.42, *r*_100_ = 0.10, *r*_140_ = 0.17; S5 – *r*_40_ = 0.31, *r*_60_ = 0.34, *r*_100_ = 0.30, *r*_140_ = 0.15.

We explored what attributes of words and sentences might further affect reading performance within the sentence reading task. For all participants, accuracy was highest for short words, suggesting that performance here may be highly influenced by working memory or word frequency effects (Figure 5C). However, accuracy was not systematically related to sentence length, suggesting that the sustained attention required to read longer sentences did not impair reading performance (Figure 5D). Across all word lengths, accuracy was significantly correlated with word frequency and this correlation remained even when short and long words (which are associated with high and low frequencies, respectively) were removed (Figure 6). Other single word linguistic measures available from the ELP database (bigram frequency, number of syllables or morphemes, number of orthographic neighbors) were not significantly correlated with reading performance (data not shown). Overall, this suggests that reading performance could be enhanced by presenting low-frequency words with longer letter durations.

### Paragraph reading with SLR

**Figure 7:**
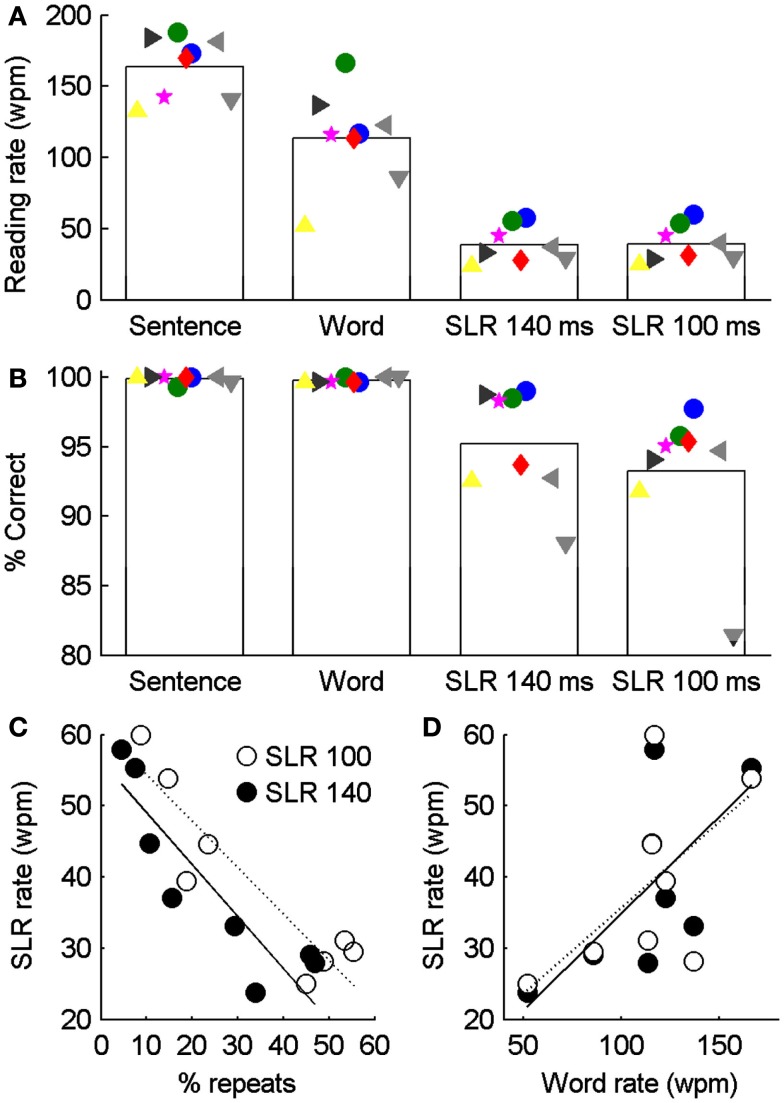
**Comparison of reading accuracy and rate for whole paragraphs presented as complete sentences, single words, or individual letters (SLR with *t*_Letter_ 140 or 100 ms)**. Bar graphs show mean word identification accuracy **(A)** and reading rate **(B)**. Large green and blue spots correspond to data from subjects S4 and S5 in earlier figures, who had extensive training from the previous tasks before completing the paragraph task. Other data points correspond to naïve subjects who had no previous exposure to single letter reading. Reading rate in the SLR conditions was significantly negatively correlated with the proportion of words that were repeatedly viewed [**(C)**: *r*_100_ = −0.91, *p*_100_ < 0.01; *r*_140_ = −0.89, *p*_140_ < 0.01], but was not significantly correlated with the reading rate of whole sentences [**(D)**: *r*_100_ = 0.43, *p*_100_ = 0.29; *r*_140_ = 0.48, *p*_140_ = 0.23].

As a final test of SLR, we tested the ability of two participants trained on the previous tasks and six naive participants to read 100–101 word short stories under four different conditions. Using stories rather than isolated words means that reading is more natural and each word has the benefit of context at both the level of the sentence and paragraph. Reading rates of 130–190 wpm were observed in a control condition in which stories were presented with whole sentences visible on the screen at once, and a keypress required to display the subsequent sentence (Figure 7A). Similar, but slower, rates of 52–167 wpm were observed in a second control condition in which only single words were visible, and a keypress was required to advance to the next word. The slower rate reflects both the continual button pressing required to view each word and also the different strategies adopted by different participants. Notably, in both the whole sentence and whole word control tasks, reading accuracies were >99% in all participants, i.e., they made fewer than three mistakes across >300 words (Figure 7B).

We compared two SLR conditions, with letter timings of 100 and 140 ms. Not surprisingly, for all participants, reading rates and accuracies were significantly reduced relative to control conditions (Figure 7). Reading rates and accuracies were highest in the two participants with prior training, but despite this, considering only the data from the naive participants, a mean reading rate of >30 wpm and accuracy >90% was achieved. While, reading rates and accuracies were not significantly different between the two SLR conditions (*t*-test, *p* > 0.05), a significantly greater number of words were repeated with the shorter, *t*_letter_ 100 ms condition than the 140 ms condition. This is also reflected by the strong negative correlation between the reading rate and the percentage of times that words were repeatedly viewed (Figure 7C). Thus while short letter presentation times allow a word to be presented more rapidly, they increase the probability that the word will need to be repeated to be identified. It was expected that readers who were slower on the control conditions would also have the lowest read rates in the SLR conditions, however, there is no significant correlation between reading rates measured with SLR and the more natural control condition of whole sentences (Figure 7D). Based on subjective observations, this may reflect different reading strategies adopted by each participant when faced with the novel task of SLR, which may be ameliorated by task-specific training.

## Discussion

We tested the ability of normally sighted participants to read words, sentences, and paragraphs presented as a sequential stream of single letters. Reading rates and accuracies were similar under reading conditions in which the rate of letter presentation was fixed or controlled by the participant. The primary determinants of accuracy were the rate at which single letters were presented, word length, and word frequency. Font size had little effect on reading accuracy. Importantly, for three participants tested extensively, with a single letter presentation time of 100 ms, reading rates of ∼60 wpm were achieved and the accuracy with which words were identified on their first viewing was 83–93% across participants. Across six naive participants tested with little training, rates of 30–60 wpm and accuracies >95% were consistently achieved.

### Minimal requirements for reading

The process of normal reading begins with word recognition and lexical access to match a word with its meaning (Coltheart et al., 2001). Thus, a primary aim of this study was to determine if word recognition and lexical access was possible with SLR. Reading comprehension is impoverished in young children and some people with alexia who adopt a letter-by-letter reading strategy (Warrington and Shallice, 1980). This reading strategy is a pathological spatial analog to the artificial reading process enforced across time by the SLR method, suggesting that even in normal adults, comprehension may not be feasible with SLR. Further, in normal reading, comprehension benefits from the spatial context of letters within words, words within sentences, and sentences within paragraphs. While it is known that high reading rates and accurate word recognition can be achieved using RSVP of whole English words (Gilbert, 1959; Forster, 1970) and Chinese characters (Zhao et al., 2011), no previous studies have examined the ability to read non-ideographic text based on the presentation of single letters at a time. In our LDT, a strong correlation was observed between word frequency and both the accuracy and reaction time for recognition of true words. Further, reaction times for rejecting pseudohomophones were longer than for correctly identifying real words. In whole word LDTs, these are hallmarks of lexical access (Balota et al., 2007), suggesting that lexical access occurs in SLR and, although not specifically tested here, comprehension is possible.

Having demonstrated that an important and minimal requirement of reading, lexical access, is met by SLR, what other perceptual and cognitive factors affect prosthetically enabled reading? Normal reading rates for adults when reading aloud can exceed 200 wpm (Legge et al., 1985), and reading rates with Braille can exceed 100 wpm (Mousty and Bertelson, 1985). However, reading rates differ depending on whether the participant is skimming, scanning, or reading attentively. For many applications of prosthetic-based reading, such as reading signs and labels, only a few words need to be accurately identified and therefore high reading rates are not critical (Whittaker and Lovie-Kitchin, 1993). For the purpose of reading longer passages of text, achieving reasonable comprehension levels without frustrating the user will probably require rates of at least 30–40 wpm (Pelli et al., 1985; Whittaker and Lovie-Kitchin, 1993). Our current data demonstrates that while this is attentionally demanding, it can easily be achieved with SLR, even in untrained participants. Further, there are likely to be significant benefits above the accuracies reported here as a result of practise (Sommerhalder et al., 2003) and with the meaningful and purposive context gained from sentences and paragraphs (Fine and Peli, 1996). For example, in our task, although there was meaningful context in the stories, participants were not tested on comprehension, and many reported that they adopted reading strategies that emphasized word identification accuracy over comprehension. Thus, while SLR is likely to meet the minimal requirements for reading comprehension, direct testing of comprehension with the purposeful reading of longer passages needs to be demonstrated.

### Limitations of previous approaches to simulated prosthetic reading

Previous approaches to simulating prosthetic reading have assumed that the way in which visual images are converted to electrical stimulation will be identical for navigation, object manipulation, and reading. Three aspects of this direct conversion may make reading difficult to achieve, and suggest that a special “reading mode” such as SLR may be advantageous. First, the contrast polarity of both digital and paper text is usually dark letters on a light background. This means that regions of text will activate the majority of electrodes in an array in order to represent the light background. This is likely to make letter identification against the background difficult. Further, activation of the majority of electrodes will maximize power consumption, and increases the likelihood of current spread and unpredictable interactions between phosphenes (Schmidt et al., 1996).

Second, there are considerable challenges associated with spatial resolution. Without a manual zoom option, there is no guarantee that text will be of the optimal size to fit within the camera’s field of view. Further, as feasible electrode arrays are limited to a few thousand electrodes, whole words cannot easily be represented (Figure 1). While text can be scanned with close-to-normal accuracy when only four letters are visible, and accurate reading is still possible when only portions of a letter are visible (Legge et al., 1985), it may be difficult to reliably and simultaneously represent multiple letters if phosphenes are not of regular size and separation. The issue of irregular phosphenes is likely to be a larger problem for cortical than retinal prostheses as grid-like electrode arrays in cortex are not associated with linear grid-like arrangements of phosphenes, and blood vessels and sulcal folding mean there may be large separations between adjacent electrode arrays, with corresponding gaps in the representation of the visual field (Dobelle et al., 1974; Schmidt et al., 1996; Bradley et al., 2005). Finally, with direct conversion of the video signal, the scanning eye or head movements required for reading will generate motion blur due to the limitations on the update rate of microstimulation (Dobelle et al., 1976; Zrenner et al., 2011). This may severely limit the scanning speed if providing a clear representation of each letter is a priority.

A prosthetic “reading mode” incorporating SLR may be an efficient method for overcoming the problems detailed above. The contrast polarity of letters against background and the spatial pattern of activated electrodes can be chosen to represent each letter with minimum overlapping phosphenes and in an energy efficient manner. Importantly, this maximizes the likelihood of identifying each letter, since they are represented using all available phosphene locations. As letter presentation is not affected by motion blur or camera movements, it is limited only by the rate at which the prosthetic stimulation can be updated and the rate at which a letter stream can be recognized by the patient. With a 10 Hz update rate (i.e., 100 ms/letter), our results suggest that a reading rate of up to 60 wpm and >95% accuracy can be achieved with minimal training. It remains to be seen if backward masking, whereby an ongoing stimulus can interfere with the perception of previously presented stimuli, is a problem with prosthetic representations (Raab, 1963). While visual letter presentation at 10 Hz was not problematic in our study, interference effects associated with backward masking may have longer time constants with electrical stimulation and a 10 Hz update rate with changing electrical stimulation may not be possible (Dobelle et al., 1976; Dobelle, 2000). Some of the problems of backward masking may be ameliorated by changing the duty cycle of stimulation, e.g., by decreasing *t*_visible_ and increasing *t*_gap_ in our paradigm.

### Practical implementation of single letter reading

We were surprised by the ability of all participants to accurately recognize words at the highest presentation rates; with *t*_letter_ = 40 ms, word recognition performance in sentences was >65% for all participants. While this demonstrates very rapid integration of letters within a word, this level of accuracy is unlikely to be sufficient to support adequate comprehension. However, it is possible that reading accuracy at very short *t*_visible_ times can be improved by lengthening *t*_gap_. Future research should more extensively investigate these intra-word timing ratios, as well as inter-word ratios (the time taken to present a word relative to the blank between words). In addition to helping optimize reading performance, these intra- and inter-word duty-cycles may be critical when reading with a prosthetic device because minimizing the electrode stimulation time (effectively *t*_visible_) is desirable to minimize power consumption. Thus, a trade-off exists in how words are presented: with ASP-Words, it is possible to obtain higher reading rates than participants adopt naturally with ESP-Words, however, this increased reading speed comes at the expense of reduced word recognition accuracy. It is possible that with extensive training, participants could improve accuracy with the fast-ASP-Words conditions (Sommerhalder et al., 2003, 2004), but in general, the ASP-Words conditions make reviewing single missed words difficult. Therefore, the most satisfying and useable condition may be ESP-Words, or perhaps some condition in which multiple words or short phrases are presented after each button press.

Although participants did not achieve 100% word recognition accuracy in the Sentence task, the results from the Paragraph task suggest this is unlikely to be a problem if words can be viewed multiple times. For example, if a 90% word recognition accuracy is achieved on the first viewing, we expect that accuracy would reach 99% with a second viewing, with obvious trade-offs in reading rate. Practically, how could a process such as SLR be implemented? We propose that text could be automatically extracted from computer based text, or else optical character recognition could be used to extract text from high resolution static images of the environment, as chosen by the patient. Such methods are already feasible, e.g., the SYPOLE project (Gaudissart et al., 2004; Peters et al., 2004). The prosthesis would then be set into a reading mode, in which single letters were robustly rendered using the majority of available electrodes and phosphenes, and with this representation stabilized with respect to subsequent object, eye, and head movements. With automated presentation, participants would need the ability to repeat words or sentences, and perhaps to adjust the letter timing. An obvious limitation of such an approach is that it requires constant interaction with the device such as a button press every second. However, normal vision is a highly active task, incorporating eye movements, focusing and attention, thus manual interaction with a prosthesis may be necessary to fully exploit its possibilities. Further, low vision readers already cope with such active methods for reading when using CCTV devices.

Some enhancements beyond SLR using fixed letter timings are relatively straightforward, and remain to be tested. For example, our analysis of the effect of word frequency within the lexicon suggests that letter presentation durations could be increased for words with low-frequency, or for words which are difficult to identify for other reasons. Some electrodes could be reserved to indicate the position of a letter within a word, a word in a sentence, or a sentence in a paragraph, thus providing spatial cues to word position and context. Future work is also required to test the feasibility of SLR with different visibility duty-cycles, with peripheral vision (Sommerhalder et al., 2003, 2004) and with spatially degraded letters that are pixelised (or phosphenised) with random noise (Cha et al., 1992; Dagnelie et al., 2006; Fornos et al., 2011). We have demonstrated that SLR with automatic presentation of letters within a word and user-elicited word presentation offers a simple method for prosthetic-based reading. Importantly, it could easily be implemented in most feasible visual prostheses, regardless of their temporal and spatial resolution.

## Conflict of Interest Statement

The authors declare that the research was conducted in the absence of any commercial or financial relationships that could be construed as a potential conflict of interest.
